# PD-L1 expression in bone marrow plasma cells as a biomarker to predict multiple myeloma prognosis: developing a nomogram-based prognostic model

**DOI:** 10.1038/s41598-020-69616-5

**Published:** 2020-07-28

**Authors:** Byung-Hyun Lee, Yong Park, Ji Hye Kim, Ka-Won Kang, Seung Jin Lee, Seok Jin Kim, Byung Soo Kim

**Affiliations:** 1 Department of Internal Medicine, Korea University College of Medicine, Anam Hospital, 73, Inchon-ro, Seongbuk-gu, Seoul, 02841 Korea; 2 Department of Biomedical Science, Graduate School of Medicine, Korea University, Seoul, Korea; 3 Department of Internal Medicine, Samsung Medical Center, Sungkyunkwan University School of Medicine, Seoul, Korea

**Keywords:** Myeloma, Prognostic markers, Translational immunology

## Abstract

PD-L1 expression is associated with poor prognosis, although this relationship is unclear in bone marrow-derived haematologic malignancies, including multiple myeloma. We aimed to determine whether PD-L1 expression could predict the prognosis of newly diagnosed multiple myeloma (NDMM). We evaluated 126 NDMM patients (83, retrospectively; 43, prospectively) who underwent bone marrow examinations. Bone marrow aspirates were analysed for PD-L1 expression, categorized as low or high expression, using quantitative immunofluorescence. High PD-L1 expression could independently predict poor overall survival (OS) (95% CI = 1.692–8.346) in multivariate analysis. On subgroup analysis, high PD-L1 expression was associated with poor OS (95% CI = 2.283–8.761) and progression-free survival (95% CI = 1.024–3.484) in patients who did not undergo autologous stem cell transplantation (ASCT) compared with those who did. High PD-L1 expression was associated with poor OS despite frontline treatments with or without immunomodulators. Thus, PD-L1 expression can be a useful prognosis predictor in NDMM patients, whereas ASCT may be used in patients with high PD-L1 expression. We developed a prognostic nomogram and found that a combination of PD-L1 expression in bone marrow plasma cells and clinical parameters (age, cytogenetics, and lactate dehydrogenase) effectively predicted NDMM prognosis. We believe that our nomogram can help identify high-risk patients and select appropriate treatments.

## Introduction

Programmed cell death ligand 1 (PD-L1) plays an important role in mediating immune response and in tumour tolerance^1^ by binding to programmed cell death-1 (PD-1) on T lymphocytes and promoting T-cell exhaustion, apoptosis, and selective suppression of tumour-specific T-cells^2^. Tumour cell expression of PD-L1 has recently been found to have intrinsic effects, such as increasing cell proliferation, migration, invasion, and drug resistance, as well as decreasing apoptosis^2^. Previous studies have shown that PD-L1 is expressed in many types of malignancies, including multiple myeloma (MM)^3–5^, and immune checkpoint blockades targeting the PD-1/PD-L1 pathway. Thus, it is a promising treatment for solid tumours and lymphomas^6–8^. However, in June 2017, two related phase three trials (NCT02576977 and NCT02579863) that were evaluating the utility of pembrolizumab (an anti-PD-1 monoclonal antibody) with immunomodulatory drugs (IMiDs: pomalidomide and lenalidomide) for relapsed/refractory MM (RRMM) and newly diagnosed MM (NDMM) were suspended. These trials were suspended because of concerns regarding immune-related toxicity in the pembrolizumab arms^9,10^. In the NCT02579863 trial, the relative risk of death in the pembrolizumab arm was more than double the risk in the control group (overall survival [OS], 13% vs. 6%); however, the overall response rates were comparable between the two arms (64% vs. 62%). In the pembrolizumab arm, non-disease progression causes of death that contributed to worse survival were identifiable, including myocarditis, Stevens-Johnson syndrome, cardiac failure, pneumonitis, multiple organ dysfunction, respiratory failure, and unknown immune-related adverse events. Furthermore, trials combining IMiDs with PD-L1 inhibitors (durvalumab and atezolizumab) or a PD-1 inhibitor (nivolumab) have also been stopped because of similar safety issues^9^. Therefore, new strategies are needed to target the PD-L1 pathway by modulating the immune response via the intrinsically aggressive characteristics of MM cells themselves as well as via the PD-1/PD-L1 interaction^2^.

Expression of PD-L1 can predict treatment resistance and unfavourable prognosis^11–13^, although it remains unclear whether this relationship is true in patients with MM^1,10^. For example, soluble PD-L1 levels predicted treatment response and progression-free survival (PFS) in NDMM patients^14^, and high levels of soluble PD-L1 in bone marrow plasma were associated with worse OS rates and worse responses after autologous stem cell transplantation (ASCT) in MM patients^15^. However, detection of soluble PD-L1 is an indirect method, and the mechanisms that generate soluble PD-L1 remain poorly understood. Nevertheless, multiple studies have demonstrated the prognostic significance of PD-L1 expression using immunohistochemistry (IHC) in solid tumours^12,16,17^ and some lymphomas^18,19^. In this context, IHC can be a reliable tool for evaluating PD-L1 expression, although it is prone to intra-observer and inter-observer variability^20^. To solve the problems associated with the interpretation of IHC, an automated quantitative analysis method has been developed using immunofluorescent dyes in compartment areas^21^ and has been broadly used for solid tumours. However, it is difficult to use this method for bone marrow-derived haematologic malignancies, which do not have clear masses or tumour boundaries.

This study aimed to quantitatively assess PD-L1 expression in bone marrow plasma cells and evaluate whether PD-L1 expression was associated with survival outcomes. Based on the results, we developed a nomogram incorporating PD-L1 expression into selected clinical parameters, and we believe that this nomogram can help identify high-risk patients with poor predicted prognoses.

## Results

### Expression of PD-L1 and clinical features

**Figure 1:**
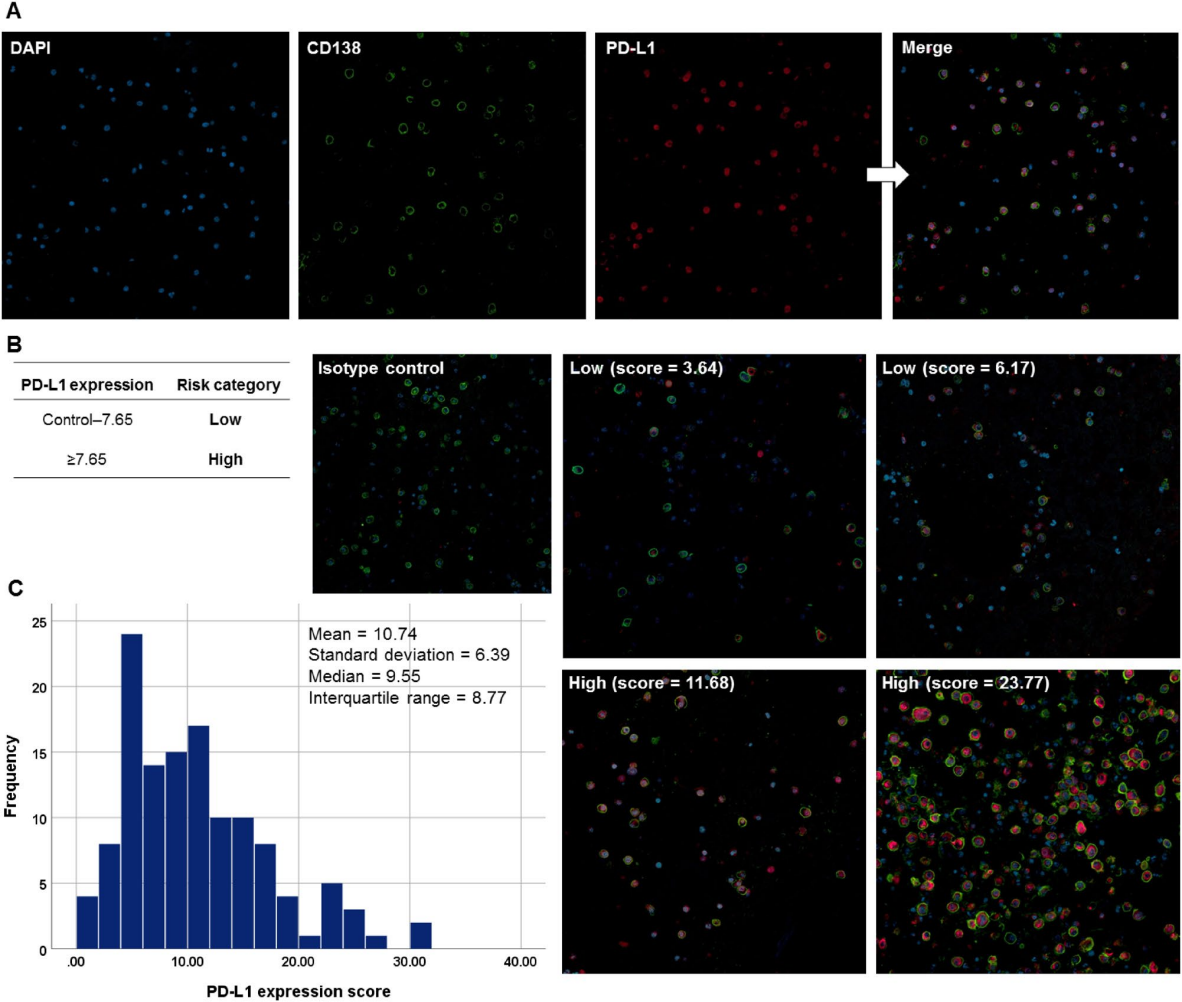
Immunofluorescence analysis of PD-L1 expression in bone marrow-aspirated plasma cells from patients with multiple myeloma. (**A**) Formalin-fixed, paraffin-embedded bone marrow aspirate specimens (clot section) from myeloma patients were sectioned at 4–5 µm. The sections were then incubated with antibodies to CD138 (1:100) and PD-L1 (1:100) overnight at 4 °C, followed by incubation with the appropriate secondary antibodies (Alexa Fluor 488, 1:200 and Alexa Fluor 647, 1:200) at room temperature for one hour. Nuclei were counterstained using DAPI, and all images were captured using a confocal laser scanning microscope (CLSM 800, Carl Zeiss Microscopy GmbH). Original magnification × 200. (**B**) Representative immunofluorescence images for the PD-L1 expression scores and groups based on the predetermined cut-off value of 7.65. (**C**) The distribution of the PD-L1 expression scores in patients with multiple myeloma. *PD-L1* programmed death-ligand 1.

We examined the entire 126 bone marrow samples for the analysis of PD-L1 expression and clinical features. Expression levels of PD-L1 were determined using the quantitative immunofluorescence (QIF) method, presented in Supplementary Fig. S1. Figure 1A shows mono-stained and merged immunofluorescence images. Figure 1B shows representative images according to the PD-L1 expression levels, and Fig. 1C shows the distribution of the PD-L1 expression in MM patients. The median and mean PD-L1 values were 9.55 (interquartile range, 5.78–14.55) and 10.74 (95% confidence interval [CI], 9.61–11.87), respectively. According to the predefined cut-off value of 7.65, 77 patients had high PD-L1 expression and 49 patients had low PD-L1 expression. Both groups had similar baseline clinical features (Table 1).

**Table 1:** Comparing the clinical features of the groups with low and high PD-L1 expression.

	Low (n = 49)	High (n = 77)	*P*
**Age, years**	67 (59.5–72.0)	66 (58.0–72.0)	
≥ 70 years	22 (44.9)	28 (36.4)	0.340
**Sex**			
Female	22 (44.9)	31 (40.3)	0.607
**ECOG performance status**			
≥ 2	1 (2.0)	7 (9.1)	0.149
**Serum M-protein, g/dL**	2.1 (0.5–4.6)	1.9 (0.4–4.0)	
≥ 3.0 g/dL	22 (44.9)	32 (41.6)	0.712
**BM plasma cells, %**	30.5 (16.4–56.5)	38.4 (20.4–70.2)	
≥ 60%	7 (8.9)	16 (20.8)	0.358
**β2-microglobulin, mg/L**	4.1 (3.1–10.2)	5.4 (3.2–8.4)	
≥ 5.5 mg/L	18 (36.7)	37 (48.1)	0.212
**Albumin, mg/dL**	3.4 (2.8–4.0)	3.2 (2.9–3.9)	
< 3.5 mg/L	26 (53.1)	45 (58.4)	0.553
**LDH, IU/L**	402.0 (299.3–485.5)	397.0 (308.5–507.5)	
≥ Upper normal range	20 (40.8)	36 (46.8)	0.513
**Cytogenetic abnormalities**			
High risk*	13 (26.5)	25 (32.5)	0.479
**ISS**			
Stage I	9 (18.4)	14 (18.2)	0.396
Stage II	22 (44.9)	26 (33.8)	
Stage III	18 (36.7)	37 (48.1)	
**R-ISS**			
Stage I	6 (12.2)	8 (10.4)	0.911
Stage II	31 (63.3)	48 (62.3)	
Stage III	12 (24.5)	21 (27.3)	
**mSMART 3.0**			
Standard	29 (59.2)	42 (54.5)	0.609
High	20 (40.8)	35 (45.5)	

Data are shown as number (percentage) or median (interquartile range).

*BM* bone marrow, *ECOG* Eastern Cooperative Oncology Group, *ISS* International Staging System, *LDH* lactate dehydrogenase, *mSMART* Mayo Stratification for Myeloma and Risk-adapted Therapy, *PD-L1*, programmed death-ligand 1, *R-ISS* Revised International Staging System.

*High-risk cytogenetics were defined as t(4;14), t(14;16), del(17/17p), TP53 deletion, or chromosome 1 abnormalities including gain(1q) and del(1p).

**Figure 2:**
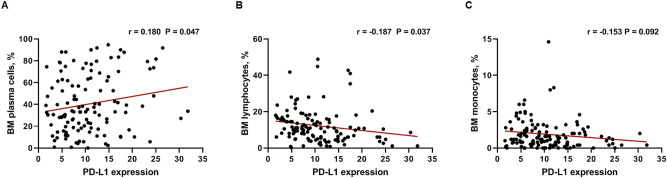
Correlation between PD-L1 expression and a percentage of bone marrow (**A**) plasma cells, (**B**) lymphocytes, and (**C**) monocytes in patients with multiple myeloma.

Then, we examined the correlation between PD-L1 expression and traditional risk factors of myeloma, including age, osteolytic lesions, ISS stage, M-spike, β2 microglobulin, LDH, and percentage of plasma cells, lymphocytes, and monocytes in bone marrow using a Pearson correlation analysis. The percentage of plasma cells in the bone marrow was significantly correlated with PD-L1 expression (r = 0.180; 95% CI 0.003–0.347; *P* = 0.047; Fig. 2A). Significant negative correlation was observed between the percentage of bone marrow lymphocytes and PD-L1 expression (r = − 0.187; 95% CI − 0.353 to − 0.011; *P* = 0.037; Fig. 2B). The percentage of bone marrow monocyte also negatively correlated with PD-L1 expression, with borderline significance (r = − 0.153, 95% CI − 0.321–0.025; *P* = 0.092; Fig. 2C). All other factors were not correlated with PD-L1 expression. Next, we evaluated the prognostic significance of PD-L1 expression according to treatment strategies.

### Clinical outcomes according to PD-L1 expression

**Figure 3:**
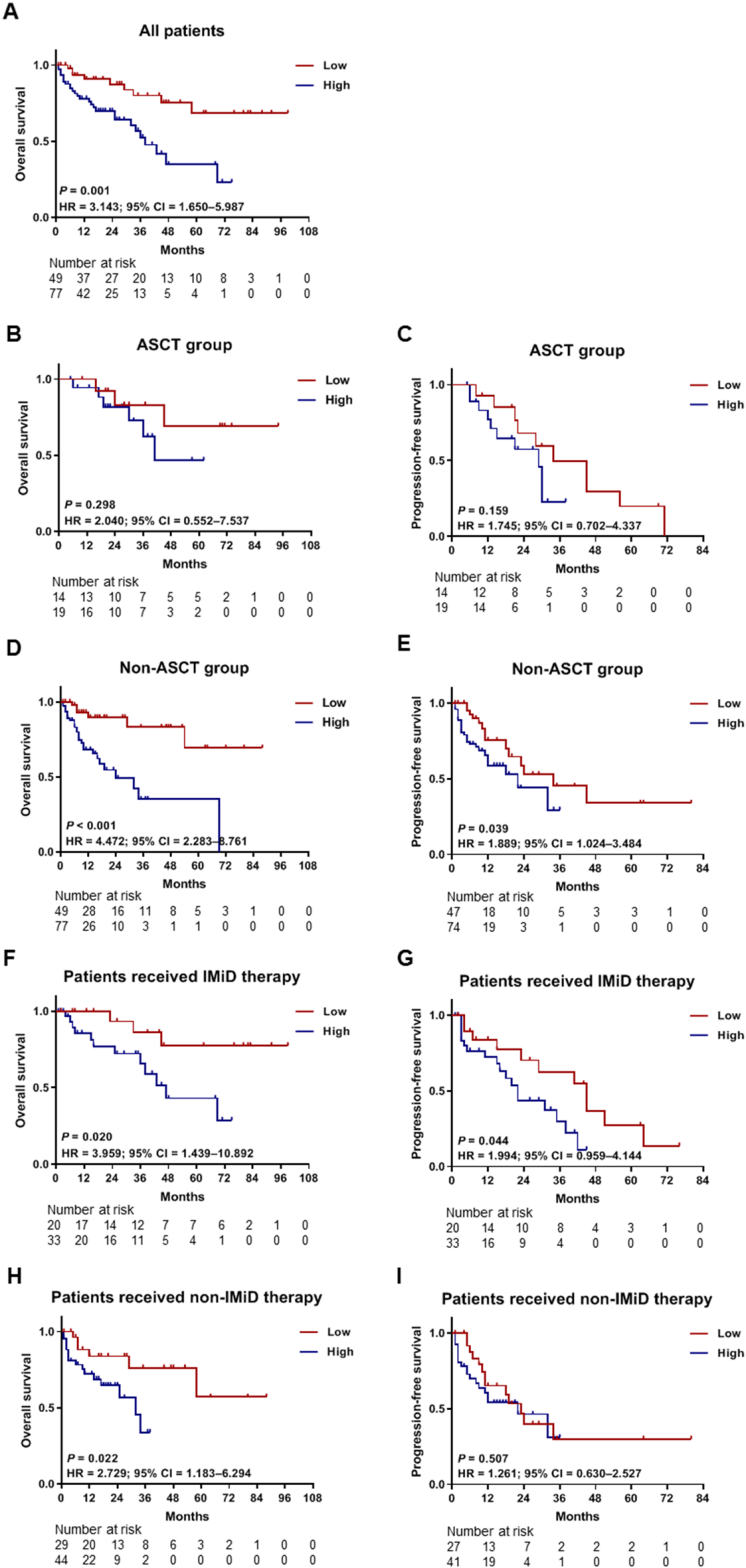
Kaplan–Meier survival curves for OS and PFS according to PD-L1 expression. PD-L1 expression values were classified as high expression (≥ 7.65) or low expression (< 7.65). (**A**) The OS curves for all patients (n = 126). The OS (**B**) and PFS (**C**) curves for the ASCT group (n = 33) are shown. The OS (**D**) and PFS (E) curves for the non-ASCT group (n = 126), including 93 patients who did not receive ASCT and 33 patients censored at the time of ASCT. Besides, the OS (**F**) and PFS (**G**) curves for the subgroups of patients (n = 53) who received frontline VTD, TD, or RD therapy that included IMiD are shown in this figure. The OS (H) and PFS (**I**) curves are for the subgroups of patients who did not receive IMiD therapy (n = 73). *ASCT* autologous stem cell transplantation, *IMiD* immunomodulatory drug, *OS* overall survival, *PFS* progression-free survival, *RD* lenalidomide-dexamethasone, *TD* thalidomide-dexamethasone, *VMP* bortezomib-melphalan-prednisolone, *VTD* bortezomib-thalidomide-dexamethasone.

We examined all 126 bone marrow samples for analysis of clinical outcomes according to PD-L1 expression. High PD-L1 expression was associated with significantly shorter OS (hazard ratio [HR], 3.143; 95% CI 1.650–5.987; *P* = 0.001; Fig. 3A). In the ASCT group (n = 33), high PD-L1 expression was not significantly associated with OS (HR, 2.040; 95% CI 0.552–7.537; *P* = 0.298; Fig. 3B) and PFS (HR, 1.745; 95% CI 0.702–4.337; *P* = 0.159; Fig. 3C). However, in the non-ASCT group (n = 126) including 93 patients who did not receive ASCT and 33 patients censored at the time of ASCT, high PD-L1 expression was associated with poor OS (HR, 4.472; 95% CI 2.283–8.761; *P* < 0.001; Fig. 3D) and PFS (HR, 1.889; 95% CI 1.024–3.484; *P* = 0.039; Fig. 3E). High PD-L1 expression was also associated with poor OS (HR, 3.959; 95% CI 1.439–10.892; *P* = 0.020; Fig. 3F) and PFS (HR, 1.994; 95% CI 0.959–4.144; *P* = 0.044; Fig. 3G) in patients who received frontline VTD, TD, or RD (bortezomib-thalidomide-dexamethasone, thalidomide-dexamethasone, or lenalidomide-dexamethasone) therapy that included IMiD. In patients who received frontline VMP (bortezomib-melphalan-prednisolone) therapy that did not include IMiD, OS (HR, 2.729; 95% CI 1.183–6.294; *P* = 0.022; Fig. 3H) and PFS (HR, 1.261; 95% CI 0.630–2.527; *P* = 0.507; Fig. 3I) were also poor regardless of PD-L1 expression status. In summary, high PD-L1 expression negatively affected the prognosis of MM patients; however, this effect was attenuated when they received ASCT. Then, to confirm the effect of PD-L1 expression in survival prognosis, we conducted univariate and multivariate analyses and included other relevant clinical factors.

### Predictors of overall survival

**Figure 4:**
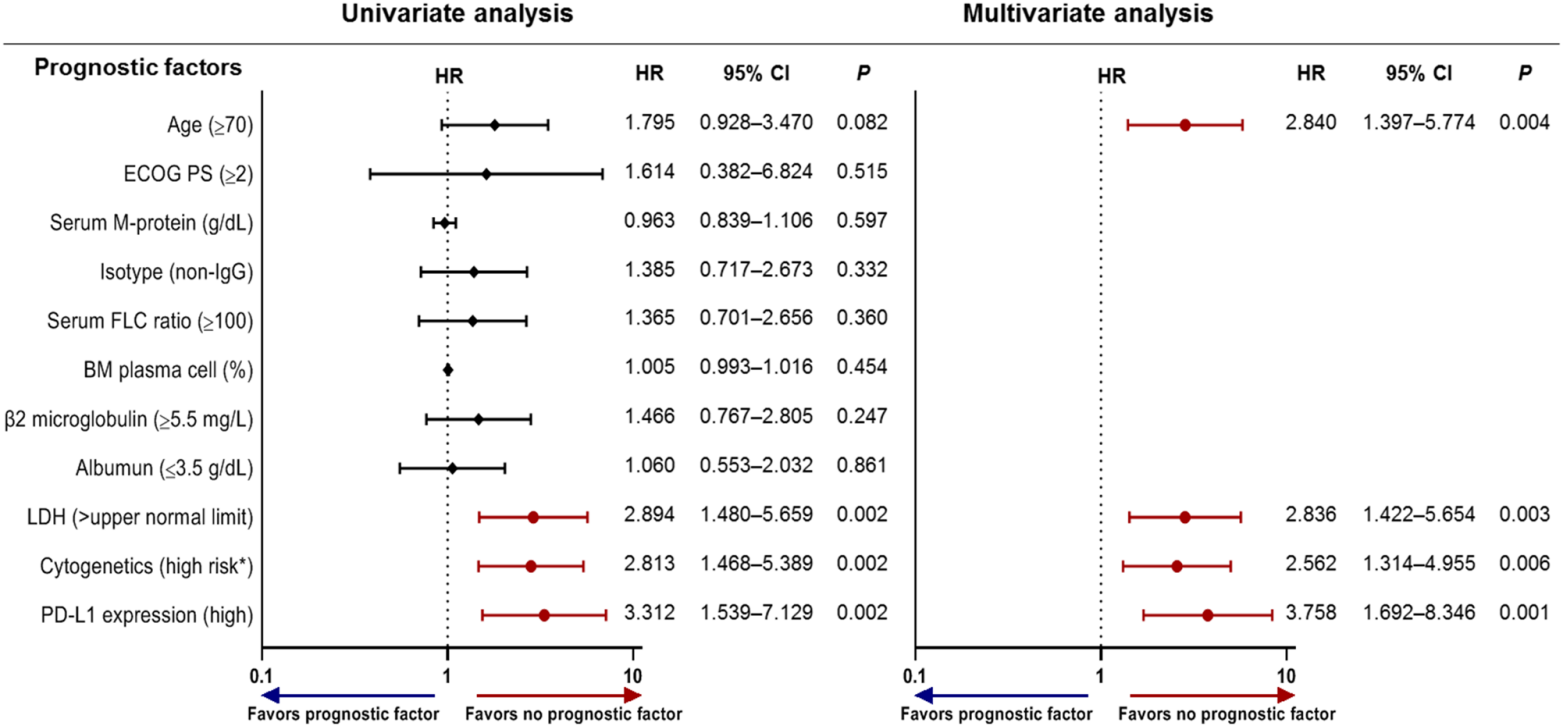
Univariate and multivariate Cox regression analyses for OS. If the hazard ratio is greater than 1, then the predictor is associated with an increased risk of death. *OS* overall survival, *BM* bone marrow, *ECOG* Eastern Cooperative Oncology Group, *LDH* lactate dehydrogenase, *PS* performance status. * High-risk cytogenetics were defined by the presence of at least one of the following: t(4;14), t(14;16), del(17/17p), TP53 deletion, and chromosome 1 abnormalities including gain(1q) and del(1p).

Figure 4 shows the results of the Cox regression analyses. We examined all 126 bone marrow samples for these analyses. The univariate analyses revealed that a poor prognosis was associated with high PD-L1 expression, lactate dehydrogenase (LDH) levels ≥ the upper normal limit (UNL), and at least one of the following high-risk cytogenetic factors: t(4;14), t(14;16), del(17/17p), TP53 deletion, and chromosome 1 abnormalities including gain(1q) and del(1p). Multivariate Cox analysis using the backward stepwise elimination method and including all variables assessed in univariate analysis (age, Eastern Cooperative Oncology Group [ECOG] performance status, serum M-protein, the isotype of immunoglobulin, serum free-light chain ratio, bone marrow plasma cells, β2-microglobulin, albumin, LDH, cytogenetic risk factors, and PD-L1 expression) confirmed that a poor prognosis was independently predicted by high PD-L1 expression (hazard ratio [HR], 3.758; 95% CI 1.692–8.346; *P* = 0.001), LDH levels ≥ UNL (HR, 2.836; 95% CI 1.422–5.654; *P* = 0.003), high-risk cytogenetic factors (HR, 2.562; 95% CI 1.314–4.955; *P* = 0.006), and age ≥ 70 years (HR, 2.840; 95% CI 1.397–5.774; *P* = 0.004). As a result, PD-L1 expression was confirmed as a significant prognostic marker in MM patients. Based on these results, we constructed a new scale to predict the prognosis of MM.

### Construction and validation of the prognostic nomogram

As shown in Supplementary Table S1, there were no significant differences between the training and validation cohorts in terms of age, ECOG performance status, serum M-protein, bone marrow plasma cells, β2-microglobulin, albumin, cytogenetic abnormalities, International Staging System (ISS), Revised ISS (R-ISS), and mSMART 3.0 classification (*P* = 0.121–0.931). However, significant differences were observed between the two cohorts in terms of serum M-proteins (*P* = 0.015), LDH levels (*P* = 0.026), and the initial treatment regimens (*P* = 0.025).

**Figure 5:**
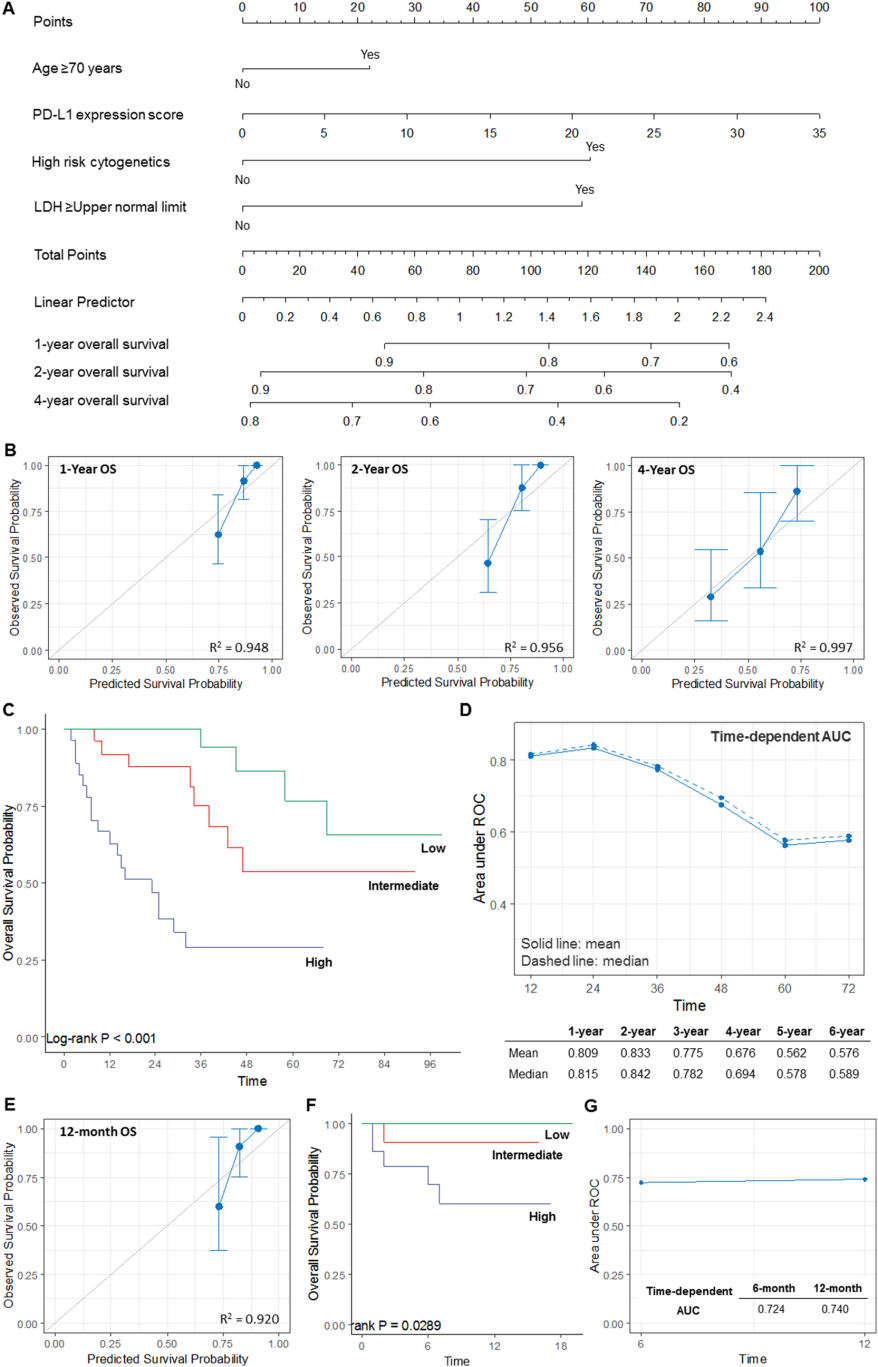
Development of the prognostic nomogram. (**A**) A nomogram for estimating OS in the training (retrospective) cohort. (**B**) Calibration curves for predicting 1-year, 2-year, and 4-year OS were created by plotting the observed survival probabilities (y-axis) against the nomogram-predicted probabilities (x-axis). The vertical bars indicate the 95% CIs, which were calculated by bootstrapping with 1,000 resamples. The 45-degree line indicates an ideal model. (**C**) The Kaplan–Meier OS curves in the three risk groups according to the tertiles of the nomogram’s total score. (**D**) Time-dependent AUC analyses with 1,000 bootstrap replicates for evaluating the performance and prediction accuracy of the nomogram in the training cohort. (**E**) Calibration curves for 12-month OS in the validation (prospective) cohort, which were created by plotting the observed survival probabilities (y-axis) against the nomogram-predicted probabilities (x-axis). The 45-degree line indicates an ideal model. (**F**) The Kaplan–Meier OS curves in the validation cohort according to the nomogram. (**G**) The time-dependent AUC analyses in the validation cohort. *AUC* area under the curve, *OS* overall survival, *CI* confidence interval, *PFS* progression-free survival.

We constructed a prognostic nomogram-based model on the scores for PD-L1 expression, LDH, cytogenetics, and age, with higher scores predicting a poorer prognosis (Fig. 5A). The 83 patients from the training (retrospective) cohort were assigned to either a low-risk group (score of < 49.8), an intermediate-risk group (score of 49.8–99.6), or a high-risk group (score of > 99.6), and these cut-offs were based on the tertile levels in the training cohort. The calibration plots for the probabilities of 1-year, 2-year, and 4-year OS showed good agreement between the observed and predicted outcomes (Fig. 5B), and significant differences in OS were observed among the three risk groups (*P* < 0.001; Fig. 5C). Time-dependent area under the curve (AUC) analyses were applied with 1,000 bootstrap replications to evaluate the performance of the nomogram (Fig. 5D). We judged the performance to be good at 1–4 years based on the mean AUC values of 0.676–0.833 and median AUC values of 0.694–0.842. When applied to the validation (prospective) cohort, the nomogram exhibited good calibration at 12 months (Fig. 5E). The 43 patients were assigned to either a low-risk, an intermediate-risk, or a high-risk group, and significant differences in OS were observed among the three groups (*P* = 0.029; Fig. 5F). The AUC of 0.740 at 12 months indicated that the performance of the model was good (Fig. 5G). After developing and validating a new prognostic model, we compared our model to R-ISS.

### Comparing the new prognostic model and the R-ISS

**Figure 6:**
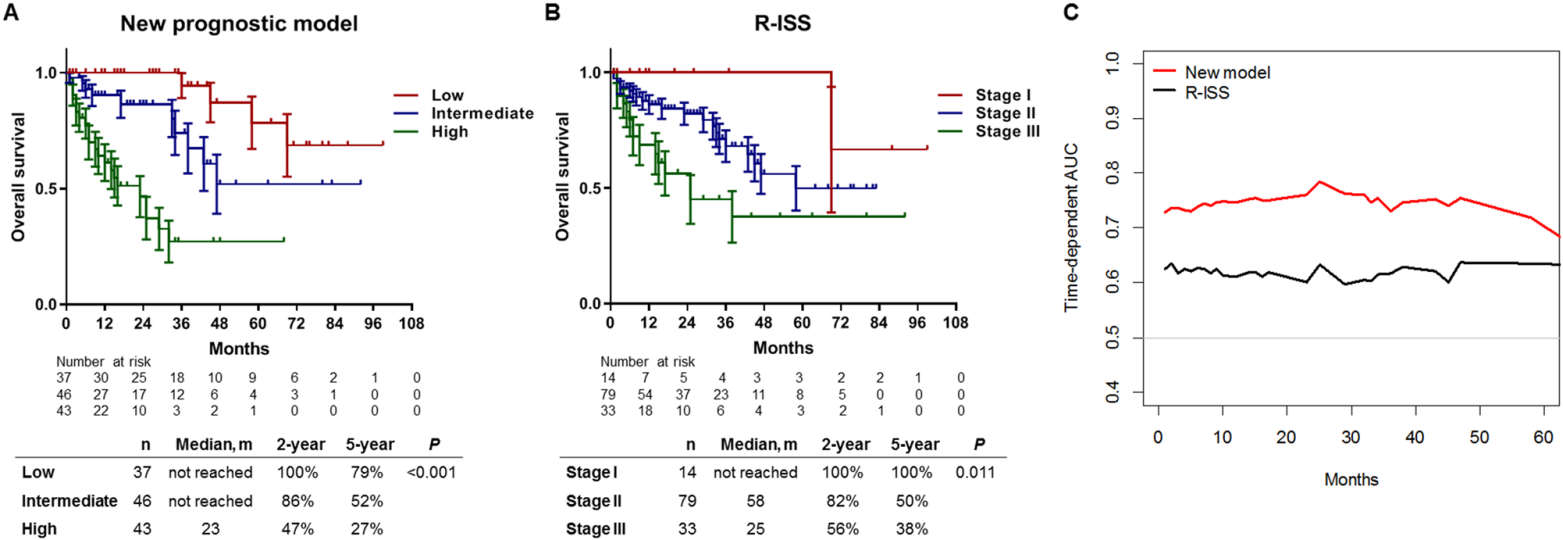
Comparison of the new prognostic model and the R-ISS. (**A**) The Kaplan–Meier OS curves with SE based on the new prediction model and (**B**) the R-ISS. (**C**) The time-dependent ROC curves and AUC analyses for OS. *AUC* area under the curve, *OS* overall survival, *R-ISS* Revised International Staging System, *ROC* receiver operating characteristic, *SE* standard error.

We conducted a comparative analysis between our model and the R-ISS and included the entire cohort of 126 patients. According to the new prognostic model, the median OS was not reached in the low-risk and intermediate-risk groups, although it was 23 months in the high-risk group (*P* < 0.001; Fig. 6A). The 2-year OS rates were 100% in the low-risk group, 86% in the intermediate-risk group, and 47% in the high-risk group. The 5-year OS rates were 79% in the low-risk group, 52% in the intermediate-risk group, and 27% in the high-risk group. According to the R-ISS, the median OS was not reached in the low-risk groups, although it was 58 months in the intermediate-risk group and 25 months in the high-risk group (*P* = 0.011; Fig. 6B). When the R-ISS was used, the 2-year OS rates were 100% in the stage I group, 82% in the stage II group, and 56% in the stage III group, while the 5-year OS rates were 100%, 50%, and 38%, respectively.

The new prognostic model provided better discriminating power than did the R-ISS, as indicated by the C-index values (0.775 [95% CI 0.717–0.833] vs. 0.650 [95% CI 0.568–0.732]; *P* = 0.005; Table 2). Time-dependent receiver operating characteristic (ROC) analysis was also used to compare the models (Fig. 6C), and the AUC values revealed that the new prognostic model had an improved value. The Net Reclassification Index was also calculated to compare the performance of the models, which revealed that 71 patients (56.3%) were reclassified using the new prognostic model (see Supplementary Table S2). Among the patients with events (death), 11 patients in the low-/intermediate-risk groups were correctly reclassified to the intermediate-/high-risk groups, while six patients were incorrectly reclassified to the low-/intermediate-risk groups. Among the patients without events (survival), 54 patients were reclassified, including 18 patients with incorrect reclassification. The Net Reclassification Index of the nomogram was determined to be 0.337, which indicated better discrimination power than that exhibited by the R-ISS.

**Table 2:** Comparing the discriminative powers of the new prognostic model and the R-ISS.

	Harrell’s C	95% CI	Difference	95% CI	*P*
New prognostic model	0.775	0.717–0.833	0.125	0.037–0.213	0.005
R-ISS	0.650	0.568–0.732			

*CI* confidence interval, *R-ISS* Revised International Staging System.

## Discussion

To the best of our knowledge, this is the first study to indicate that high PD-L1 expression in bone marrow-aspirated plasma cells is associated with a poor prognosis in NDMM. Our subgroup analyses revealed that ASCT was associated with an improved prognosis in the high PD-L1 expression group, although it did not prevent the progression or relapse of NDMM. Based on this result, we created a nomogram-based new prognostic model using PD-L1 expression and various clinical characteristics to stratify patients into three risk groups. In addition, the nomogram was able to accurately identify patients who were likely to have a poor prognosis. New therapeutic strategies are needed to improve the survival and cure rates of patients in the high-risk group.

Patients with MM exhibit PD-L1 expression in their plasma cells^22–24^, and patients with persistent minimal residual disease have high expressions of PD-L1 and PD-1^25^. Thus, PD-L1 expression may have prognostic value in MM, although there is controversy regarding the appropriate method for measuring PD-L1 expression in individual bone marrow samples, and the reported data are affected by the use of different antibody clones and detection methods^26^. For example, a clinical study of pembrolizumab, pomalidomide, and low-dose dexamethasone in RRMM examined PD-L1 expression in bone marrow biopsy specimens using IHC^27^. In that study, the PD-L1 expression levels were not correlated with the treatment outcomes. The PD-L1 cut-off used in that study was similar to the cut-off value used for lung cancer cases, although it is unclear whether this approach is suitable for MM cases. Therefore, an accurate method is needed to evaluate PD-L1 expression and to determine whether an appropriate cut-off value can help guide the prognostication of MM.

The common method for evaluating PD-L1 expression is IHC, and there are various commercially available anti-PD-L1 clones and IHC tests for solid cancers. For example, the PD-L1 IHC 22C3 pharmDx assay is approved by the Food and Drug Administration for selective PD-1/PD-L1-targeted immunotherapy (pembrolizumab) in non-small-cell lung cancer. However, the positivity thresholds and scoring systems vary according to the anti-PD-L1 antibody and cancer type, and there is no consensus regarding the optimal antibody, detection method, and PD-L1 positivity cut-off for MM. This may be related to the difficulty in distinguishing between scattered tumour cells and other bone marrow components in trephine biopsy samples from bone marrow-derived malignancies, such as MM, especially compared with the relatively clear boundary between the adjacent-infiltrating cells and solid cancers or lymphomas. The decalcification process for trephine biopsy samples can also affect the staining intensity and IHC findings, which cannot be corrected using image analysis^28^. Thus, we hypothesised that QIF using bone marrow-aspirated specimens might aid in more accurately evaluating PD-L1 expression while avoiding issues related to inter-observer and intra-observer variability and/or the decalcification process. A similar approach has been used for solid cancers, with the QIF score for PD-L1 expression in the tumour and adjacent stroma being calculated by dividing the sum of the target PD-L1 intensities in the tumour compartment pixels by the sum of the compartment pixel areas^21^. In this study, we modified this approach by calculating the QIF score using PD-L1 expression in individual plasma cells and then dividing that value by the total number of gated plasma cells. This cell gating method is not complicated, can readily be performed using available image-processing software, and is not affected by the decalcification of the bone marrow aspirate samples. These properties may help standardise the evaluation of PD-L1 expression in bone marrow aspirate samples, especially relative to the variable methods that are currently used with different antibody clones, detection methods, and scoring algorithms.

There is no approved clones and methods for detection of PD-L1 in multiple myeloma. Thus, we tested our method using PD-L1 22C3 (DAKO) antibody approved for diagnostic assay for pembrolizumab treatment and 28–8 (Abcam) antibody that a same clone from a different vendor (DAKO) approved for diagnostic assay for nivolumab treatment. Further information can be found in the Supplementary Fig. S2. Because 28–8 (DAKO) is only available for pharmDx kit, we have used 28–8 (Abcam) antibody. PD-L1 levels using 22C3 antibody were concordant with those used in our study (see Supplementary Fig. S3). Although IHC 28–8 pharmDx test is approved diagnostic assay for nivolumab treatment in non-small cell lung cancer and melanoma, expression of 28–8 antibody did not detected in the setting of our study. Considering our method is based on immunofluorescence (IF) analysis, expression of PD-L1 by IF method could be different from IHC method. Further studies could be required to comparing expression patterns of multiple PD-L1 antibodies in multiple myeloma and optimizing detection methods and protocols. In addition, we compared PD-L1 levels obtained from the new quantification method to those from flow cytometry. The results showed that PD-L1 levels by the new quantification method were correlated with those obtained by flow cytometry (see Supplementary Fig. S4).

The efficacy of the PD-1 blockade is associated with the mutational burden and effector cell infiltration into the tumour bed, which are lower in MM than in solid tumours^2^. Given that the effects of PD-L1 on tumour physiology are mediated via intrinsic effects on the tumour cells, as well as extrinsic binding to PD-1 on effector cells, it may be useful to selectively treat high-risk MM patients according to their PD-L1 expression levels, regardless of their PD-1 status. For example, we found that patients with high PD-L1 expression experienced improved OS and PFS after ASCT, although this did not prevent disease progression, and half of the patients ultimately died within 41 months. Moreover, high PD-L1 expression was independently associated with poor OS (HR, 3.758; 95% CI 1.692–8.346; *P* = 0.001). Some previous studies have examined the significance of soluble PD-L1 in MM^14,15^, although soluble PD-L1 levels do not reflect the intrinsic effects of PD-L1 on cell proliferation, apoptosis, migration, and drug resistance^2^. Thus, the clinical relevance of soluble PD-L1 remains unknown, as the origin and the exact function of soluble PD-L1 remain unclear. It is possible that soluble PD-L1 might neutralise the effects of PD-L1 antibodies or induce hypersensitivity reactions^29^. Therefore, evaluating the expression of PD-L1 in the bone marrow using QIF may be more useful for prognostication and treatment selection, relative to evaluating soluble PD-L1.

In this study, the PD-L1 expression status did not affect OS (median, not reached vs. 41 months; HR, 2.040; 95% CI 0.552–7.537; *P* = 0.298) or PFS (median, 34 vs. 29 months; HR, 1.745; 95% CI 0.702–4.337; *P* = 0.159) in patients who received ASCT. These results contradicted the independent associations of PD-L1 expression with OS and PFS in patients who did not receive ASCT. Compared with low PD-L1 expression, high PD-L1 expression was significantly associated with unfavourable OS and PFS in patients treated with both frontline IMiD and non-IMiD regimens, although patients who received non-IMiD regimens showed poor PFS regardless of the PD-L1 expression status. Considering that our data showed that percentages of bone marrow effector cells (lymphocytes and monocytes) were negatively correlated with PD-L1 expression, immune restoration after ASCT could affect the outcome of patients with high PD-L1 expression. Thus, ASCT might be a promising therapy for patients with high PD-L1 expression, although these patients are still expected to experience progression or recurrence. A previous study reported that blockade of the PD-L1 pathway in MM might enhance the efficacy of stem cell transplantation with cell-based vaccination^30^. Therefore, strategies that combine PD-L1 inhibitors with ASCT might help improve early management strategies for and outcomes in patients with high PD-L1 expression. Further studies are needed to address the role of ASCT in association with PD-L1 expression in MM patients.

Nomogram-based models are generally more accurate than risk group–based models and may include continuous variables that are not categorised^31^. Statistical formulae could include more information than a nomogram,however, using statistical formulae is not convenient in clinical settings. We developed the nomogram using the Lasso method to avoid overfitting from the small sample size and to enhance the model’s accuracy and interpretability. The Lasso method selected age, cytogenetics, LDH level, and PD-L1 expression, which were also selected in the multivariate Cox analysis. Furthermore, the model had good calibration, as indicated by the expected and observed survival probabilities as well as the good performance and accuracy in the training and validation cohorts as per the time-dependent AUC values. Given that the R-ISS is the most common prognostic tool for NDMM^32^, we compared the accuracy and discrimination power of our nomogram to those of the R-ISS, which revealed that our nomogram had a higher C-index value (Table 2) in this study setting. Furthermore, the time-dependent AUC values revealed that our nomogram was superior for predicting OS (Fig. 6C). In our cohort, the median OS for patients with R-ISS stage III disease was 25 months, which was shorter than the value of 43 months obtained in the International Myeloma Working Group^32^, and only 14 patients (11.1%) were classified as having stage I disease. However, it is possible that real-world outcomes may be different from those predicted using the R-ISS, based on the data from 11 clinical trials^32^. Moreover, relative to the R-ISS, our nomogram was superior for predicting the OS and may be more useful for predicting early death in NDMM.

This study has several limitations. First, there is controversy regarding the most appropriate anti-PD-L1 antibody, testing method, and scoring system for evaluating PD-L1 expression in the bone marrow samples from MM patients. This study used the anti-PD-L1 clone ABM4E54, although this clone is not commercially available. Thus, it will be important to establish standardised methods for evaluating PD-L1 expression in MM. Second, this study included a relatively small sample, and the follow-up period for the validation cohort was short (18 months). Long-term and multi-centre data are needed to validate our nomogram and to determine whether it can be used to effectively select optimal strategies for treating NDMM. Third, most patients received thalidomide as IMiD, as lenalidomide was not available in our clinic for frontline therapy until 2018 due to its unavailability in the Korean National Health Insurance system. We included all patients treated with lenalidomide in the study period – a total of three. Thus, the type of IMiD might have affected survival outcomes for the high-risk patients classified by our nomogram. Additional analyses are needed to evaluate patients who received lenalidomide-based treatment.

In conclusion, this study revealed that QIF may be a useful method for evaluating PD-L1 expression in bone marrow aspirate samples and that high PD-L1 expression in bone marrow plasma cells was associated with a poor prognosis in patients with NDMM. Furthermore, we developed a prognostic nomogram model based on PD-L1 expression and several clinical factors. Combining PD-L1 expression with clinical parameters could improve the prognostic evaluation of NDMM patients. Our nomogram may help physicians identify high-risk patients and select appropriate treatment strategies.

## Methods

### Patient cohorts

This study evaluated two cohorts of NDMM patients who underwent bone marrow aspiration at the Korea University Anam Hospital (see Supplementary Fig. S5). The first retrospective cohort involved 92 consecutive NDMM patients (January 2011 to April 2018), and the second prospective cohort included 44 NDMM patients (May 2018 to October 2019). Of all 136 patients, 10 patients were excluded because bone marrow blocks were not available for them. Thus, this study examined 126 bone marrow specimens (83 from the retrospective cohort and 43 from the prospective cohort). The 83 specimens from the retrospective cohort were included in the training cohort for developing the nomogram model, and the 43 specimens from the prospective cohort were included in the validation cohort for validating the nomogram model. We conducted power analyses for determining the minimal sample size of the study population and appropriate number of samples were used for this study model. Further details can be obtained from the Supplementary Information. In the subgroup analyses, the ASCT group included patients who received ASCT during the study period and 33 patients were included. The non-ASCT group was included 93 patients who did not received ASCT during the study period and 33 patients who received ASCT during the study period censored at the time of transplantation to avoid bias from excluding these patients; thus, a total of 126 patients were included. The protocol of the study was approved by the Institutional Review Board of the Korea University Medical Center. The patients provided written informed consent to participate in this study. All methods were performed in accordance with the relevant guidelines and regulations.

### Immunofluorescence staining

The formalin-fixed paraffin-embedded specimens were cut into 4–5 µm sections, placed on slides, deparaffinised and then rehydrated. Antigen retrieval was performed by boiling in a pressure cooker for 10 min with a sodium citrate buffer (pH 6.0), and permeabilisation was achieved using 0.5% triton X-100. The specimens were blocked using 5% normal donkey serum for one hour at room temperature and then incubated overnight at 4 °C with the primary antibodies targeting CD138 (1:100; R&D Systems, Minneapolis, MN, USA) and PD-L1 (ABM4E54, 1:100; Abcam, Cambridge, UK). The samples were then incubated with the fluorochrome-conjugated secondary antibodies for the CD138 test (Alexa Fluor 488, 1:200; Invitrogen, Carlsbad, CA, USA) and the PD-L1 test (Alexa Fluor 647, 1:200; Invitrogen, Carlsbad, CA, USA) at room temperature for one hour. Isotype-matched antibodies were used as negative controls, and the nuclei were stained using DAPI mounting medium (ProLong Diamond Antifade Mountant with DAPI; Invitrogen, Carlsbad, CA, USA).

### Quantification of PD-L1 expression

All slides were assessed under 200 × magnification using a confocal laser scanning microscope (CLSM 800; Carl Zeiss Microscopy GmbH, Oberkochen, Germany), with image acquisition and subsequent analysis of 2–10 fields (median, 5 fields). All images were obtained under identical conditions, including identical bit depth (8 bit), pinhole size (1 AU), laser power, and exposures in the individual fluorescence channels. The plasma cell areas were identified by creating a cell mask using the CD138 signal. Supplementary Fig. S1 shows the process for quantifying PD-L1 expression, which was based on the mean fluorescent intensity (MFI) within each plasma cell compartment and was determined using an image-processing software (Celleste Image Analysis Software; Invitrogen, Carlsbad, CA, USA). After subtracting the background intensity, a semi-quantitative immunofluorescence score for PD-L1 expression was calculated by dividing the sum of the MFIs in all plasma cell compartments by the total number of plasma cells. The obtained MFI was then normalised by dividing it with the MFI from the isotype-matched control. According to the method proposed by Contal and O’Quigley^33^, the optimal cut-off point was identified as the point at which the absolute value of the log rank statistic was maximal. Thus, the value of the continuous variable (PD-L1 expression) gave the maximum difference between the subjects in the two groups defined by the cut-off point. We classified PD-L1 expression values as high expression (normalised MFI of ≥ 7.65) or low expression (normalised MFI of < 7.65).

### Nomogram development and validation

A prognostic nomogram was developed using the least absolute shrinkage and selection operator (Lasso) regression method^34^ based on training cohort. Leave-one-out cross-validation was used to correct for potential overfitting. The nomogram’s calibration curves were assessed by plotting the observed survival fraction against the nomogram-predicted probability based on bootstrapping with 1,000 resamples. Using tertiles derived from the total cohort training scores (retrospective), the patients were divided into three risk groups (low-, intermediate-, and high-risk groups). Time-dependent AUC analysis with 1,000 bootstrap replicates was used for validating and evaluating the nomogram’s performance. Additional validation was performed in the validation (prospective) cohort using AUC analysis.

The nomogram’s prognostic value was compared with the R-ISS based on its concordance index (C-index), which is a measure of the discriminating power for survival models. Values of < 0.5 indicate no useful discrimination, and a value of 1.0 indicates a perfect separation of patients with different outcomes^35^. Time-dependent ROC curves and AUC analyses were also used to evaluate the performance of the models.

### Statistical analysis

Categorical variables were evaluated using the chi-square test or Fisher’s exact test, and continuous variables were evaluated using the Student’s t-test or the Mann–Whitney U test. The OS was calculated from the time of diagnosis to death from any cause, and the PFS was calculated from the start of treatment to disease progression or death. In subgroup analysis for patients who received ASCT, OS was defined as the time between the start of ASCT and death from any cause, whereas PFS was defined as the time from the start of ASCT to disease progression or death. Differences in OS and PFS were evaluated using the Kaplan–Meier analysis and the log rank test. Cox proportional hazard models were used to analyse the associations between the survival outcomes and various prognostic factors. All tests were two-sided, and *P*-values < 0.05 were considered significant. The statistical analyses were performed using the R software (version 3.5.2)^36^, SPSS statistics version 25.0 software (IBM Corporation, New York, NY, USA), and GraphPad Prism software (version 8.2.1,GraphPad Software Inc., San Diego, CA, USA). The nomogram was developed using the R package ‘hdnom’ (version 5.0)^37^, and the ROC curves were compared between the new model and the R-ISS using the R package ‘risksetROC’ (version 1.0.4)^38^.

## Supplementary information

Supplementary file1

## Data Availability

The datasets generated during and/or analysed during the current study are available from the corresponding author upon reasonable request.

## References

[1] Rosenblatt J, Avigan D (2017). Targeting the PD-1/PD-L1 axis in multiple myeloma: a dream or a reality?. Blood.

[2] Tremblay-LeMay R, Rastgoo N, Chang H (2018). Modulating PD-L1 expression in multiple myeloma: an alternative strategy to target the PD-1/PD-L1 pathway. J. Hematol. Oncol..

[3] Tamura H (2013). Marrow stromal cells induce B7–H1 expression on myeloma cells, generating aggressive characteristics in multiple myeloma. Leukemia.

[4] Greaves P, Gribben JG (2013). The role of B7 family molecules in hematologic malignancy. Blood.

[5] Khunger M (2017). Programmed cell death 1 (PD-1) ligand (PD-L1) expression in solid tumors as a predictive biomarker of benefit from PD-1/PD-L1 axis inhibitors: a systematic review and meta-analysis. JCO Precis. Oncol..

[6] Reck M (2016). Pembrolizumab versus chemotherapy for PD-L1-positive non-small-cell lung cancer. N. Engl. J. Med..

[7] Kwong YL (2017). PD1 blockade with pembrolizumab is highly effective in relapsed or refractory NK/T-cell lymphoma failing l-asparaginase. Blood.

[8] Xu-Monette ZY, Zhou J, Young KH (2018). PD-1 expression and clinical PD-1 blockade in B-cell lymphomas. Blood.

[9] Krauss AC (2018). FDA analysis of pembrolizumab trials in multiple myeloma: Immune related adverse events (irAEs) and response. J. Clin. Oncol..

[10] Jelinek T, Paiva B, Hajek R (2018). Update on PD-1/PD-L1 inhibitors in multiple myeloma. Front Immunol..

[11] Kiyasu J (2015). Expression of programmed cell death ligand 1 is associated with poor overall survival in patients with diffuse large B-cell lymphoma. Blood.

[12] Wang Q, Liu F, Liu L (2017). Prognostic significance of PD-L1 in solid tumor: an updated meta-analysis. Med. (Baltimore).

[13] de Vicente JC, Rodriguez-Santamarta T, Rodrigo JP, Blanco-Lorenzo V, Allonca E, Garcia-Pedrero JM (2019). PD-L1 expression in tumor cells is an independent unfavorable prognostic factor in oral squamous cell carcinoma. Cancer Epidemiol. Biomark. Prev..

[14] Wang L (2015). Serum levels of soluble programmed death ligand 1 predict treatment response and progression free survival in multiple myeloma. Oncotarget..

[15] Huang SY (2016). Soluble PD-L1: a biomarker to predict progression of autologous transplantation in patients with multiple myeloma. Oncotarget..

[16] Parra ER (2016). Image analysis-based assessment of PD-L1 and tumor-associated immune cells density supports distinct intratumoral microenvironment groups in non-small cell lung carcinoma patients. Clin. Cancer Res..

[17] Yokoyama S (2016). Prognostic value of programmed death ligand 1 and programmed death 1 expression in thymic carcinoma. Clin. Cancer Res..

[18] Four M (2017). PD1 and PDL1 expression in primary central nervous system diffuse large B-cell lymphoma are frequent and expression of PD1 predicts poor survival. Hematol. Oncol..

[19] Goodman A, Patel SP, Kurzrock R (2017). PD-1-PD-L1 immune-checkpoint blockade in B-cell lymphomas. Nat. Rev. Clin. Oncol..

[20] Callea M, Pedica F, Doglioni C (2016). Programmed death 1 (PD-1) and its ligand (PD-L1) as a new frontier in cancer immunotherapy and challenges for the pathologist: state of the art. Pathologica..

[21] DiVito KA, Berger AJ, Camp RL, Dolled-Filhart M, Rimm DL, Kluger HM (2004). Automated quantitative analysis of tissue microarrays reveals an association between high Bcl-2 expression and improved outcome in melanoma. Cancer Res..

[22] Liu J (2007). Plasma cells from multiple myeloma patients express B7–H1 (PD-L1) and increase expression after stimulation with IFN-γ and TLR ligands via a MyD88-, TRAF6-, and MEK-dependent pathway. Blood.

[23] Dhodapkar MV (2015). Prospective analysis of antigen-specific immunity, stem-cell antigens, and immune checkpoints in monoclonal gammopathy. Blood.

[24] Yousef S (2015). Immunomodulatory molecule PD-L1 is expressed on malignant plasma cells and myeloma-propagating pre-plasma cells in the bone marrow of multiple myeloma patients. Blood Cancer J..

[25] Paiva B (2015). PD-L1/PD-1 presence in the tumor microenvironment and activity of PD-1 blockade in multiple myeloma. Leukemia.

[26] Udall M (2018). PD-L1 diagnostic tests: a systematic literature review of scoring algorithms and test-validation metrics. Diagn. Pathol..

[27] Badros A (2017). Pembrolizumab, pomalidomide, and low-dose dexamethasone for relapsed/refractory multiple myeloma. Blood.

[28] Gertych A (2014). Effects of tissue decalcification on the quantification of breast cancer biomarkers by digital image analysis. Diagn. Pathol..

[29] Gu D, Ao X, Yang Y, Chen Z, Xu X (2018). Soluble immune checkpoints in cancer: production, function and biological significance. J. Immunother. Cancer..

[30] Hallett WH, Jing W, Drobyski WR, Johnson BD (2011). Immunosuppressive effects of multiple myeloma are overcome by PD-L1 blockade. Biol. Blood Marrow Transpl..

[31] Kattan MW (2008). Should I use this nomogram?. BJU Int..

[32] Palumbo A (2015). Revised international staging system for multiple myeloma: a report from international myeloma working group. J. Clin. Oncol..

[33] Contal C, O'Quigley J (1999). An application of changepoint methods in studying the effect of age on survival in breast cancer. Comput. Stat. Data An..

[34] Tibshirani R (1997). The lasso method for variable selection in the Cox model. Stat Med.

[35] Harrell FE, Lee KL, Mark DB (1996). Multivariable prognostic models: Issues in developing models, evaluating assumptions and adequacy, and measuring and reducing errors. Stat. Med..

[36] R Core Team. R: A language and environment for statistical computing. R Foundation for Statistical Computing, Vienna, Austria. https://www.R-project.org/ (2018).

[37] Nan Xiao, Qing-Song Xu, and Miao-Zhu Li. hdnom: Building Nomograms for Penalized Cox Models with High-Dimensional Survival Data. bioRxiv, 065524 (2016).

[38] Patrick J. Heagerty and packaging by Paramita Saha-Chaudhuri. risksetROC: Riskset ROC curve estimation from censored survival data (2012).

