# Supplementary material for: PD-L1 expression in bone marrow plasma cells as a biomarker to predict multiple myeloma prognosis: developing a nomogram-based prognostic model
Source: Sci Rep. 2020 Jul 28;10:12641. doi: 10.1038/s41598-020-69616-5 (PMC7387472; doi:10.1038/s41598-020-69616-5)
Supplement: Supplementary file 1 — Supplementary file1 [file 41598_2020_69616_MOESM1_ESM.pdf]

PD-L1 Expression in Bone Marrow Plasma Cells as a Biomarker to Predict Multiple Myeloma Prognosis: Developing a Nomogram-Based Prognostic Model

Byung-Hyun Lee,[1] Yong Park,[1] Ji Hye Kim,[2] Ka-Won Kang,[1] Seung Jin Lee,[2] Seok Jin Kim[3], and Byung Soo Kim[1]*

[1]Department of Internal Medicine, Korea University College of Medicine, Anam Hospital, Seoul, Korea

[2]Department of Biomedical Science, Graduate School of Medicine, Korea University, Seoul, Korea

[3]Department of Internal Medicine, Samsung Medical Center, Sungkyunkwan University School of Medicine, Seoul, Korea

***Corresponding Author:**

Byung Soo Kim, Department of Internal Medicine, Korea University College of Medicine, Anam Hospital, 73, Inchon-ro, Seongbuk-gu, Seoul 02841, Korea

Phone: 02-920-5713,

E-mail: kbs0309@korea.ac.kr.

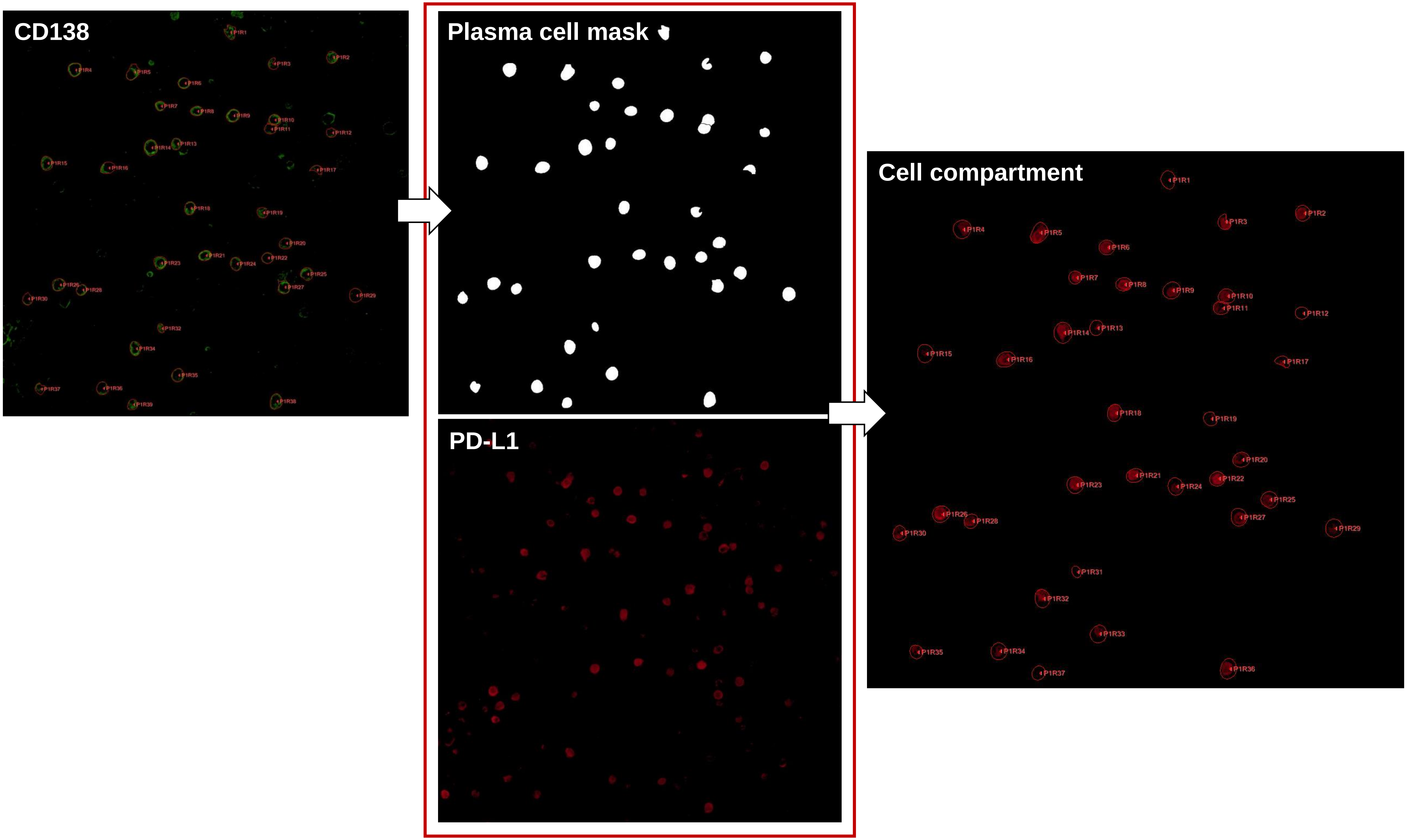

Supplementary Figure 1. Quantification process of PD-L1 expression. A plasma cell mask was created by using the CD138 signal to define the plasma cell compartment. After subtracting the background intensity, a semiquantitative immunofluorescence score for PD-L1 expression was calculated by dividing the sum of the mean fluorescence intensity (MFI) within each plasma cell compartment by the total number of plasma cells. The obtained MFI was normalized by dividing it with the MFI of an isotype-matched control. The PD-L1 expression score was determined on a scale of 0–255. All images were captured using a confocal laser scanning microscope (LSM 800, Carl Zeiss Microscopy GmbH) and analyzed using Celleste Image Analysis Software (Invitrogen). Original magnification ×200.

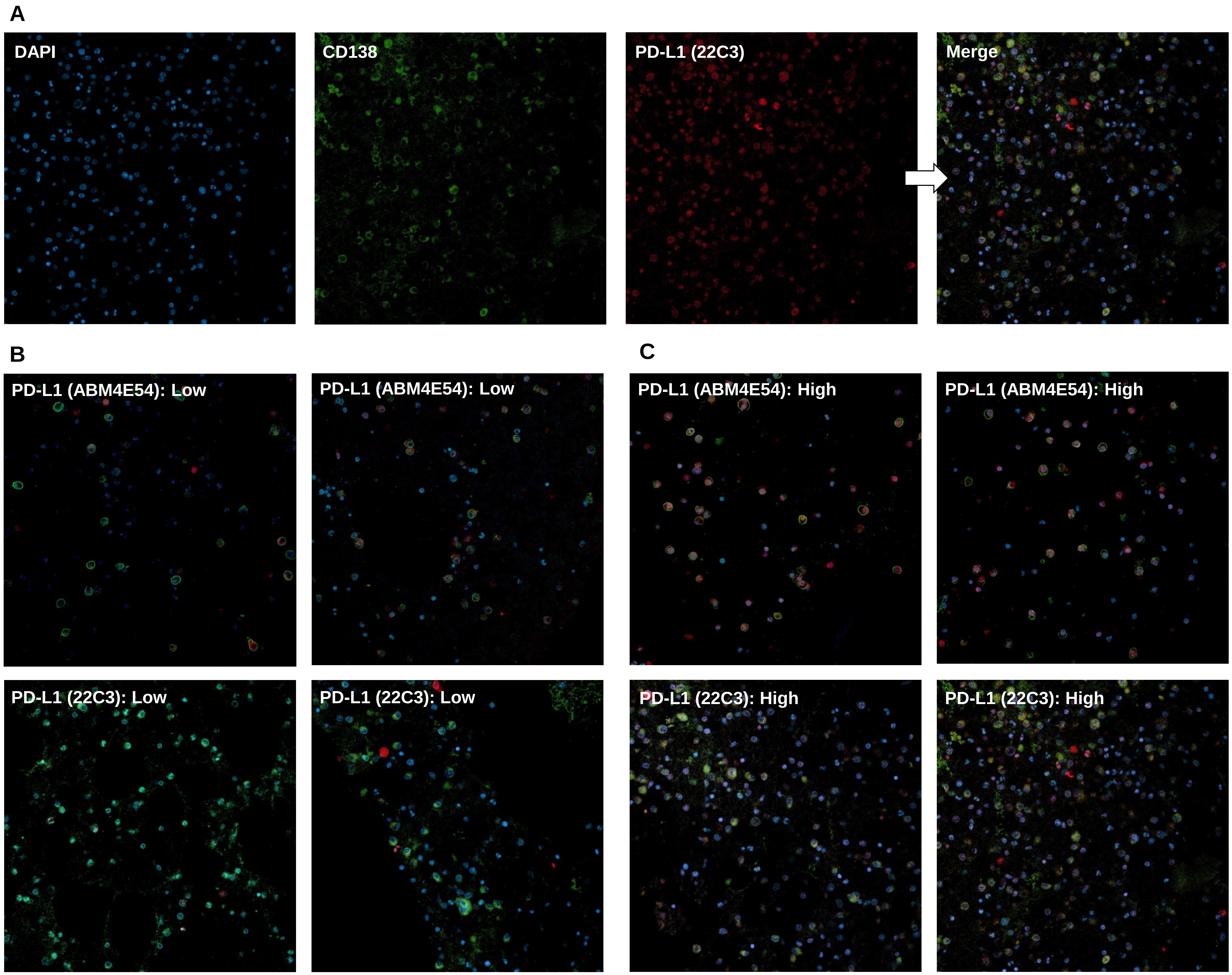

Supplementary Figure 2. Immunofluorescence analysis of PD-L1 expression using PD-L1 22C3 (DAKO) antibody approved for diagnostic assay for pembrolizumab use. (A) Formalin-fixed, paraffin-embedded bone marrow aspirate specimens (clot section) from myeloma patients were sectioned at 4–5 µm. The sections were then incubated with antibodies to CD138 (1:100) and PD-L1 (1:50) overnight at 4°C, followed by incubation with the appropriate secondary antibodies (Alexa Fluor 488, 1:200 and Alexa Fluor 647, 1:200) at room temperature for one hour. Nuclei were counterstained using DAPI, and all images were captured using a confocal laser scanning microscope (CLSM 800, Carl Zeiss Microscopy GmbH). Original magnification ×200. Representative immunofluorescence images for (B) low and (C) high PD-L1 expression comparing two PD-L1 antibody clones (ABM4E54 and 22C3).

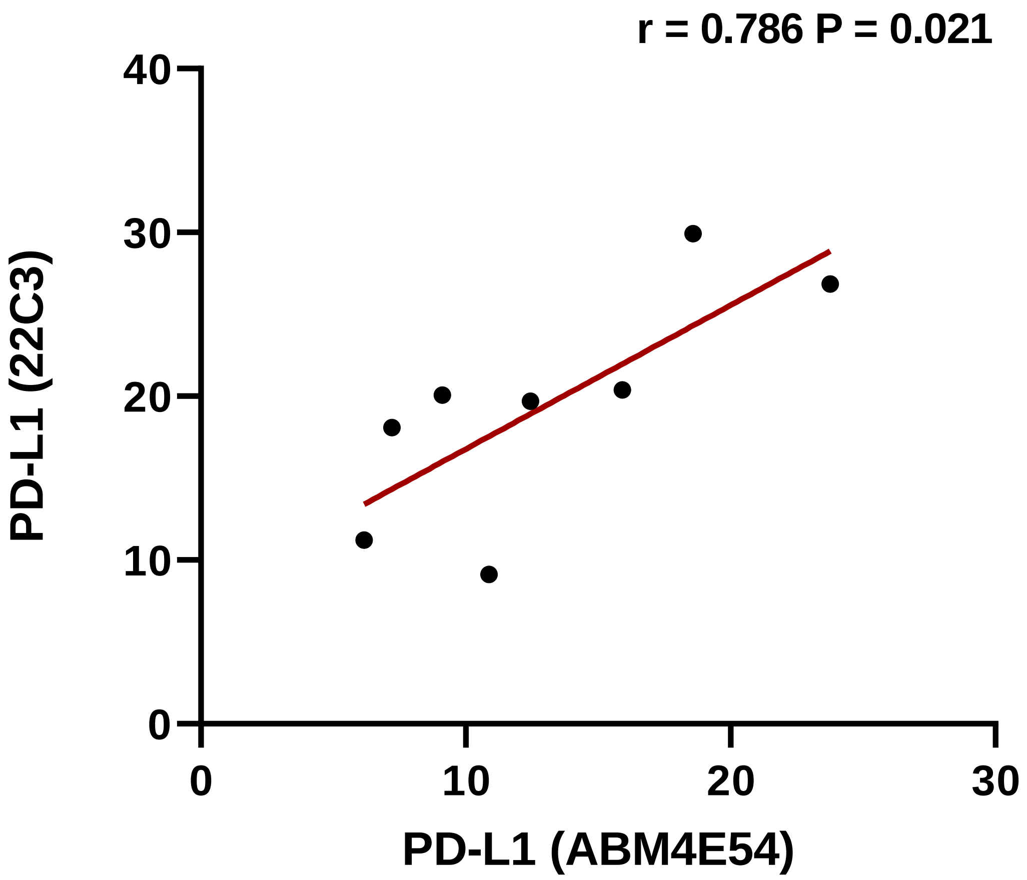

Supplementary Figure 3. Correlation of PD-L1 expression between 22C3 and ABM4E54 clones. Correlation analysis was conducted using Spearman correlation. PD-L1 levels using 22C3 antibody were concordant with those used in our study

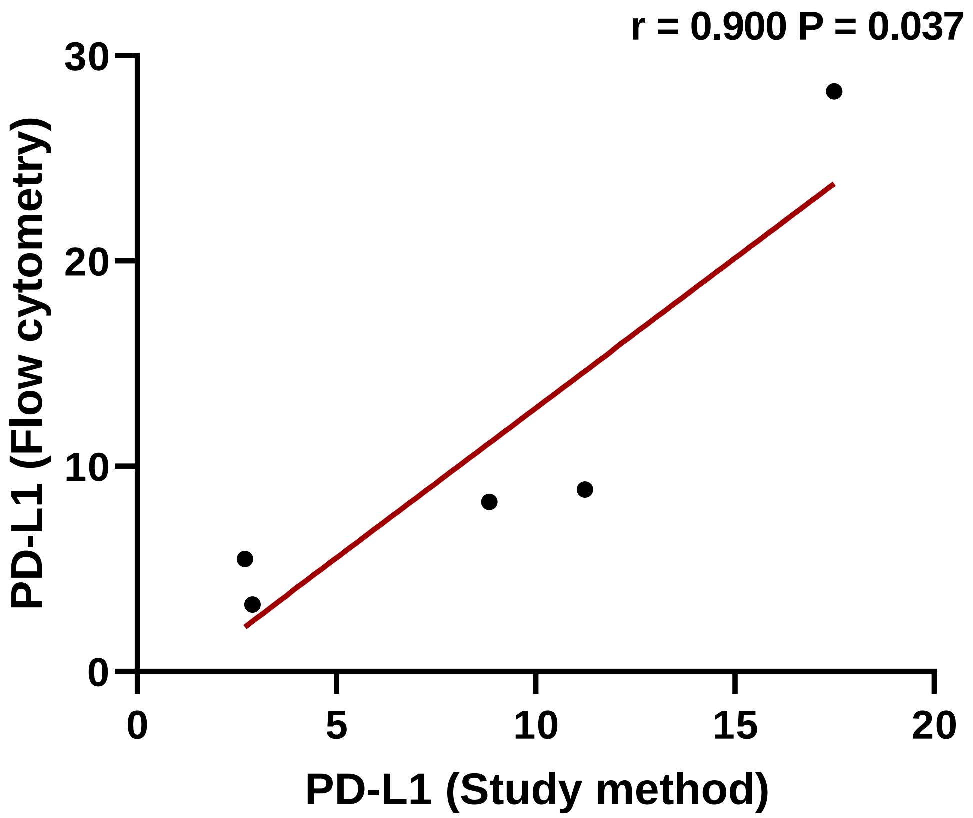

Supplementary Figure 4. Correlation of PD-L1 expression between the new quantification method and flow cytometry**.** Correlation analysis was conducted using Spearman correlation. PD-L1 levels by the new quantification method were significantly correlated with those by flow cytometry.

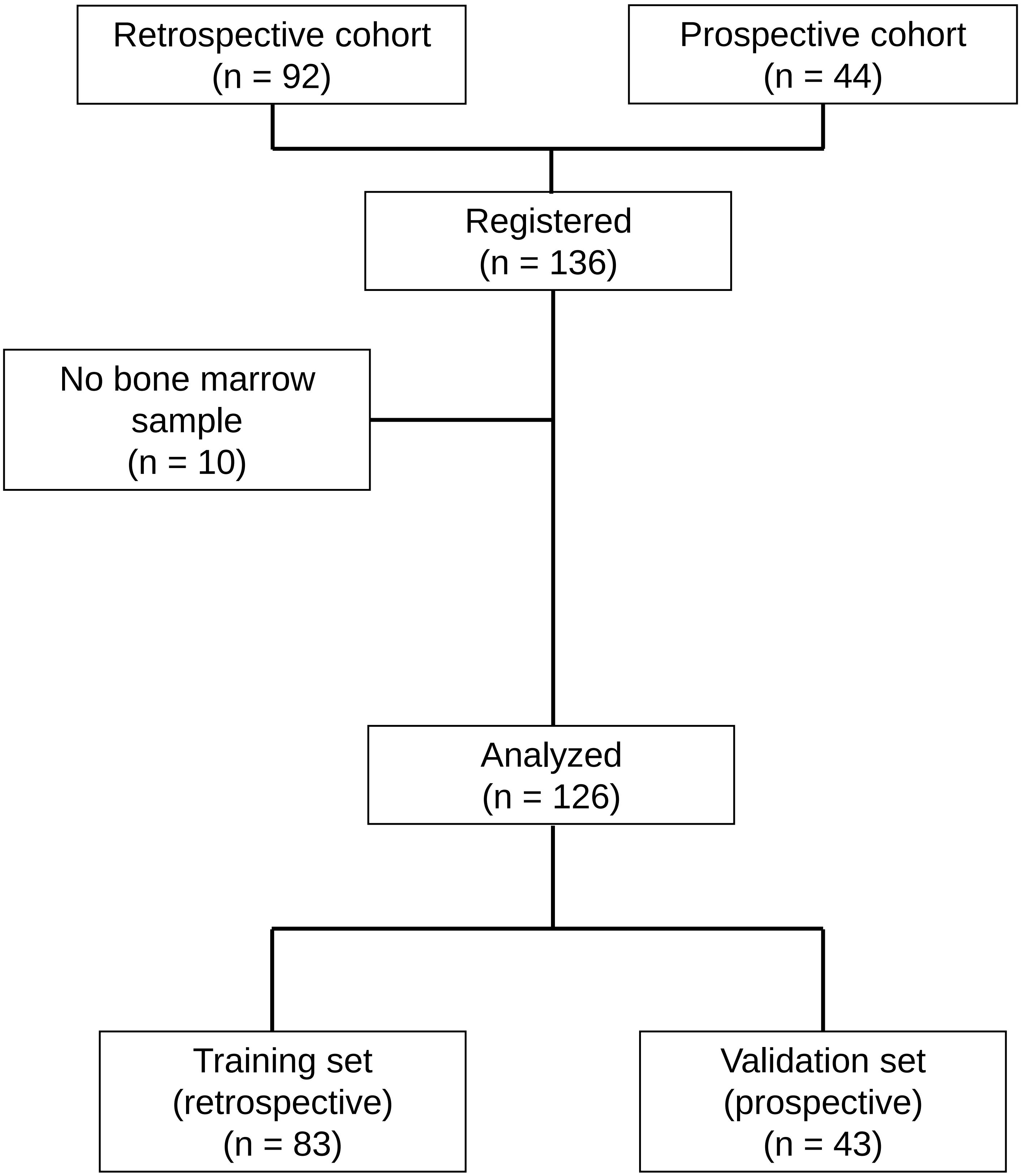

Supplementary Figure 5. Flow diagram of 136 patients registered on the study.

Supplementary Table 1. Patients characteristics of the retrospective and prospective cohorts

Characteristics	Total (n = 126)	Retrospective (n = 83)	Prospective (n = 43)
**Age, years**	66 (59–72)	66 (57–71)	68 (63–75)
≥70 years	50 (39.7)	29 (32.9)	21 (48.8)
**ECOG performance status**			
≥2	8 (6.3)	3 (3.6)	5 (11.6)
**Serum M-protein, g/dL**	2.1 (0.5–4.1)	3.1 (0.5–4.6)	1.2 (0.4–3.1)
≥3.0 g/dL	54 (42.9)	42 (50.6)	12 (27.9)
**BM plasma cell, %**	33.8 (18.8–63.2)	38.4 (22.0–64.8)	32.8 (13.6–62.2)
≥60%	23 (18.3)	16 (19.3)	7 (16.3)
**ß2-microglobulin, mg/L**	4.8 (3.1–8.8)	5.3 (3.3–8.7)	4.3 (2.8–9.7)
≥5.5 mg/L	55 (43.7)	40 (48.2)	15 (34.9)
**Albumin, mg/dL**	3.3 (2.8–3.9)	3.3 (2.9–3.9)	3.3 (3.6–3.9)
<3.5 mg/L	71 (56.3)	47 (56.6)	24 (55.8)
**LDH, IU/L**	397.0 (303.5–498.0)	379.5 (286.5–480.3)	460.0 (315.0–524.0)
≥Upper normal range	56 (44.4)	31 (37.3)	25 (58.1)
**Cytogenetic abnormalities**			
High risk*	38 (30.2)	27 (32.5)	11 (25.6)
**Initial treatment regimen**			
IMiD (VTD+TD+RD)	53 (42.1)	39 (47.0)	14* (32.6)
non-IMiD (VMP)	68 (54.0)	44 (53.0)	24 (55.8)
Supportive care only	5 (4.0)	0 (0.0)	5 (11.6)
**ISS**			
Stage I	23 (18.3)	12 (14.5)	11 (25.6)
Stage II	48 (38.1)	31 (37.3)	17 (39.5)
Stage III	55 (43.7)	40 (48.2)	15 (34.9)
**R-ISS**			
Stage I	14 (11.1)	8 (9.6)	6 (14.0)
Stage II	79 (62.7)	53 (63.9)	26 (60.5)
Stage III	33 (26.2)	22 (26.5)	11 (25.6)
**mSMART 3.0**			
Standard	71 (56.3)	45 (54.2)	26 (60.5)
High	55 (43.7)	38 (45.8)	17 (39.5)

Supplementary Table 2. Reclassification of the patients by the new prognostic model and the R-ISS

**Total**	**R-ISS**				
	Stage I	Stage II	Stage III	Total	***P***
**New predictive model**					
Low	11 (78.6%)	26 (32.9%)	0 (0.0%)	37 (29.4%)	<0.001
Intermediate	3 (21.4%)	27 (34.2%)	16 (48.5%)	46 (36.5%)	
High	0 (0.0%)	26 (32.9%)	17 (51.5%)	43 (34.1%)	

**Event (Death)**	**R-ISS**			
	Stage I	Stage II	Stage III	Total
**New predictive model**				37
Low	1	3	0	
Intermediate	0	7	3	
High	0	11	12	

**Non-event (Survival)**	**R-ISS**			
	Stage I	Stage II	Stage III	Total
**New predictive model**				89
Low	10	23	0	
Intermediate	3	20	13	
High	0	15	5	

NRI (Net Reclassification Improvement) = 0.337

Supplementary Information

For the training cohort, in a previous study, for linear models, such as multiple regression, a minimum of 10 to 15 observations per predictor variable will generally allow good estimates [pmid:15184705]. Peduzzi et al. have published simulation studies suggesting that logistic and survival models will produce reasonably stable estimates if the limiting sample size allows a ratio of approximately 10 to 15 observations per predictor [pmid:8970487]. In this study, there were 4 predictors used for prediction model and a minimum of 40 to 60 sample size should be required. The sample size of training cohort is 83, which is more than 40 to 60. For the validation cohort, we have used following formula reported by Shein-Chung Chow et al [Chow S, Shao J, Wang H. 2008. Sample Size Calculations in Clinical Research. 2nd Ed. Chapman & Hall/CRC Biostatistics Series].

$$n = \frac{1}{p_A p_B p_E}\left(\frac{z_{1-\alpha/2} + z_{1-\beta}}{\ln(\theta) - \ln(\theta_0)}\right)^2$$

$$1 - \beta = \Phi\left(z - z_{1-\alpha/2}\right) + \Phi\left(-z - z_{1-\alpha/2}\right),\ z = (\ln(\theta) - \ln(\theta_0))\sqrt{n p_A p_B p_E}$$

where, n is the sample size for validation cohort, Φ is the standard normal distribution function, α is Type I error, β is Type II error, 1-β is power, θ is the hazard ratio, pE is the overall probability of the event occurring within the study period, pA and pB are the proportions of the sample size allotted to the groups. In our study, the overall probability of the event (OS) occurring in the pilot experiment was 0.37 (pE, 31/83, the training data is used as a pilot experiment), the hazard ratio was 7.12 and pA was 0.33. Thus, the minimum number of validation samples was 34 with the desired two-sided significance level α = 0.05 and power 1-β = 90%. In our study, the validation cohorts included 43 patients, which is more than the minimum number 34.
